# Genetic Introgression and Species Boundary of Two Geographically Overlapping Pine Species Revealed by Molecular Markers

**DOI:** 10.1371/journal.pone.0101106

**Published:** 2014-06-30

**Authors:** Defang Zhang, Tao Xia, Maomao Yan, Xiaogang Dai, Jin Xu, Shuxian Li, Tongming Yin

**Affiliations:** The Southern Modern Forestry Collaborative Innovation Center, Nanjing Forestry University, Nanjing, China; USDA Forest Service, United States of America

## Abstract

Gene introgression and hybrid barriers have long been a major focus of studies of geographically overlapping species. Two pine species, *Pinus massoniana* and *P. hwangshanensis*, are frequently observed growing adjacent to each other, where they overlap in a narrow hybrid zone. As a consequence, these species constitute an ideal system for studying genetic introgression and reproductive barriers between naturally hybridizing, adjacently distributed species. In this study, we sampled 270 pine trees along an elevation gradient in Anhui Province, China and analyzed these samples using EST-SSR markers. The molecular data revealed that direct gene flow between the two species was fairly low, and that the majority of gene introgression was intermediated by backcrossing. On the basis of empirical observation, the on-site distribution of pines was divided into a *P. massoniana* zone, a hybrid zone, and a *P. hwangshanensis* zone. STRUCTURE analysis revealed the existence of a distinct species boundary between the two pine species. The genetic boundary of the hybrid zone, on the other hand, was indistinct owing to intensive backcrossing with parental species. Compared with *P. massoniana*, *P. hwangshanensis* was found to backcross with the hybrids more intensively, consistent with the observation that morphological and anatomical characteristics of trees in the contact zone were biased towards *P. hwangshanensis*. The introgression ability of amplified alleles varied across species, with some being completely blocked from interspecific introgression. Our study has provided a living example to help explain the persistence of adjacently distributed species coexisting with their interfertile hybrids.

## Introduction

Hybridization, or the crossing of different species, subspecies or ‘races’, profoundly influences species evolution. On the one hand, introgressive hybridization can promote gene flow between species, leading to the generation of new genotype combinations and thereby increasing species diversity and ecological adaptability [1]–[3]. Excessive interspecific hybridization, however, will eventually result in genetic assimilation between species [4], [5]. On the other hand, various intrinsic or extrinsic reproductive barriers that reduce hybrid fitness can be formed through introgressive hybridization, thus contributing to the maintenance of species integrity [6]. A balance between gene flow and hybrid barriers is believed to maintain the hybrid zones [7], [8] that develop when hybridization occurs between species with different environmental adaptations [9]. Environmental heterogeneity can lead to a ‘mosaic’ structure in the hybrid zone [10]–[12], as genotype selection often depends on habitat attributes.

*Pinus massoniana* Lamb and *P. hwangshanensis* Hsia are two closely related species [13]. They differ in morphology, cytology and timber anatomical characteristics [14], and also display distinct spatial separation. *Pinus hwangshanensis* is usually distributed above 1,000 m, whereas *P. massoniana* is commonly found at elevations below 700 m. An elevation range of 700 to 1,000 m is thus empirically considered to be the contact zone between these two species [15]. Trees in the contact zone possess intermediate morphological characteristics, and were at first erroneously identified as a new species [16]. Later, studies involving anatomical characterization [14], molecular markers [17] and organellar DNA [18] led to their reclassification as introgressive hybrids.

Natural hybridization is commonly observed between different pine species [19]–[21]. Similar to other pine species, pollen of *P. hwangshanensis* and *P. massoniana* is wind-dispersed, while their seeds are disseminated by animals [13]. *Pinus hwangshanensis* and *P. massoniana* have adjacent distributions, and the two species frequently overlap along a narrow contact zone. Seed plumpness, germination rates and weight per thousand seeds have been found to be significantly lower in trees within the contact zone than elsewhere [22]. A previous study revealed that morphological and anatomical characteristics of trees in the contact zone were biased towards *P. hwangshanensis* [23]. Contrary to this observation, a RAPD marker study demonstrated that gene flow was more intense from *P. massoniana* to *P. hwangshanensis* [17]. To resolve this controversy, we studied genetic introgression in these two species along an elevation gradient in their natural distributional range. In addition, we investigated whether a distinguishable boundary exists between the two species at the molecular level.

## Materials and Methods

**Figure 1:**
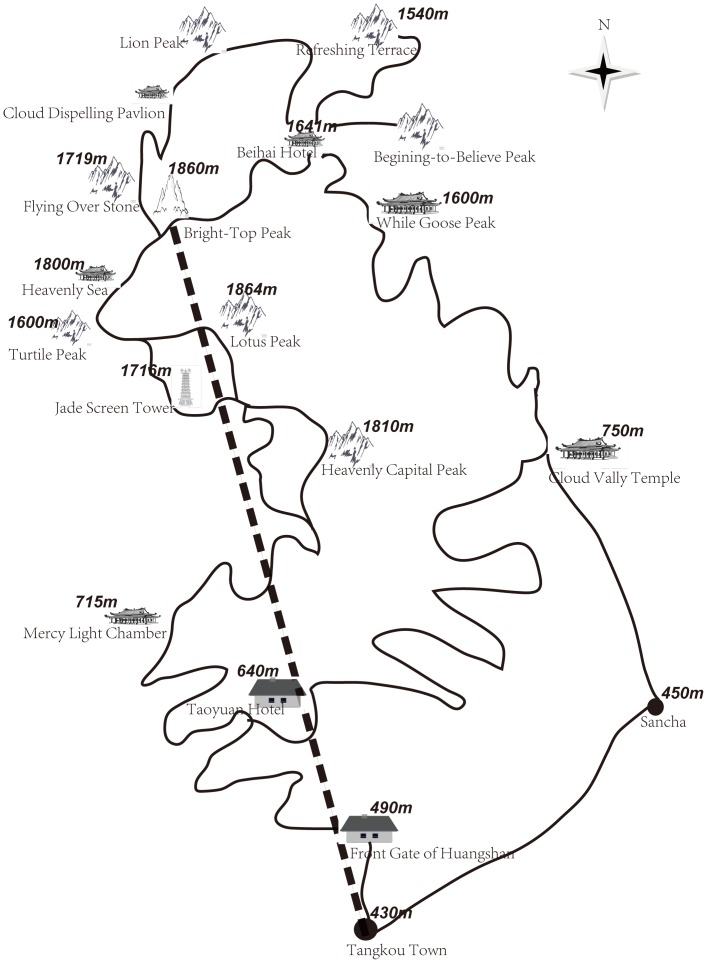
Overview of the Huangshan Mountain sampling strategy. Note: The transect line (dotted line) started at the town of Tangkou (430 m) and ended at Bright Top Peak (1,820 m).

Samples used in this study were collected from Huangshan Mountain in Huangshan, Anhui Province, China. A transect line was set up beginning in the town of Tangkou and ending at Bright Top Peak. Elevations ranged from 450 to 1,820 m (Fig 1). Needles were collected from trees located within 50 m of the transect line. In total, young needles from 270 trees were sampled from the foot to the top of the mountain in 2010. The elevation of each tree was recorded using a ZhengCheng-300 receiver (UniStrong, Beijing, China). We also recorded the breast diameter of each tree (Supporting Information S1). The field studies did not involve any endangered or protected species, and sample collection was authorized by the local administration agency. Total DNA was isolated from the sampled needles using a modified cetyltrimethylammonium bromide (CTAB) protocol [24].

Microsatellites in pine ESTs were selected from the database established by Yan et al. [25]. A total of 300 primer pairs were designed using Primer Premier 5.0 (Premier Biosoft International, Palo Alto, CA) and synthesized by Jerry Bio-Technology, Shanghai, China. Using eight DNA templates, we screened the primer pairs to identify those pairs generating distinct and highly polymorphic bands. PCR amplification, genotyping electrophoresis and data collection were performed following the protocols described in Yin *et al*. [26]. To ascertain the function of the microsatellite-containing ESTs, the corresponding sequences were searched against the NCBI non-redundant (Nr) protein database using BLASTX [27] with an E-value cut-off of 10^−5^.

### Data analyses

Drawing on a previous study of cone morphology and seed characteristics of pine trees from the same Huangshan Mountain sampling area [22], we classified the distribution of our sampled trees into three zones: a *P. massoniana* zone at elevations below 800 m, a *P. hwangshanensis* zone at elevations above 1,050 m, and a contact zone corresponding to intermediate elevations. Using POPGEN32 [28], we calculated the number of polymorphic loci (NPL), the percentage of polymorphic loci (PPL), the observed number of alleles (*Na*), the effective number of alleles (*Ne*) and Shannon's information index (*I*) for samples in each species zone. Using the genotyping data, observed heterozygosity (*Ho*) and mean expected heterozygosity (*He*) were calculated separately for each SSR marker and for samples in each species zone. Coefficients of inbreeding (*F_IS_*) and genetic differentiation (*F_ST_*) were calculated for samples in each species zone using FSTAT2.9.3 [29]. Gene flow (*Nm*) was estimated based on Wright's equation [30], *Nm* = (1−*F_ST_*)/4*F_ST_*; selfing rate (*S*) was estimated according to the formula *S* = 2*F_IS_*/(1+*F_IS_*) [31].

To analyze elevation variation in genetic structure and allele frequencies, we divided the transect into nine intervals. Each interval contained approximately 30 samples, and ranged in size from 100 to 200 m depending on tree distribution. Sample genetic structure in each elevation interval was analyzed using STRUCTURE v2.3.3 [32], [33], which estimated the natural logarithm of the probability (*Pr*) of the observed genotypic array (*X*) and calculated a pre-defined number of clusters (*K*) in the data set (ln *Pr*[*X*/*K*]) under the assumption of Hardy-Weinberg and linkage equilibrium [33]. For the STRUCTURE analysis, a Markov chain was run for 2,000,000 iterations after a burn-in of 1,000,000 iterations for values of *K* from 1 to 10, with five replicates for each *K* value. The values of the posterior probability of *K* and membership probabilities (*Q*) were recorded for each sample. Variation in allele frequencies among the nine elevation intervals was calculated using FSTAT 2.9.3 [29], followed by graphing in EXCEL.

### Data Archiving Statement

The raw data underlying the main results of this study, including primer information, sample information and the genotyping data matrix, are archived on our lab website at http://115.29.234.170/Database/Pine.

## Results

### Genetic parameters associated with each primer pair

In this study, we synthesized and screened 300 SSR primer pairs. Of the tested pairs, 14 generated distinct, highly polymorphic bands (Table 1) and were consequently used to monitor allelic variation between the two studied pine species. A total of 56 alleles were generated, with a mean number of alleles (MNA) per locus of 4 (Table 2). *Ho* varied dramatically among loci, ranging from 0.0149 to 0.9419, and *He* accordingly varied from 0.2177 to 0.7538 (Table 2). *F_ST_* and *Nm* also differed greatly among loci. The highest level of gene flow, *Nm* = 251.4594, was observed at loci genotyped by Primer30, and the lowest level was that of Primer149, with *Nm* = 0.6237 (Table 2). This broad range of values suggests that genetic introgression varied dramatically among different loci.

**Table 1:** Information on the 14 EST-SSR primers used in this study.

Locus	Primer sequence 5'-3'	Repeat type	The expected size	EST ID
Primer30	F: CTTCACATTCAACGCTGGCTAC	[TAC]_10_	165	DT625446
	R: CACTATACTGACCCTTACAATTCTTCA			
Primer33	F: CGCTATGACCTTTCGTGTT	[TCTTT]_4_	193	FE522689
	R: AATCTATGCCCCAAATTCTT			
Primer75	F: TGAGAATGCGTTTCAAAGGTGTAAGC	[CTT]_8_	144	AM982824
	R: GGTTGGCGGAAGCAGCAGAGT			
Primer89	F: GAGTCGTGGGATTTACATTCT	[AT]_7_	248	FE518792
	R: ATAGCGATTACAGGGTTGC			
Primer149	F: AGCGATGGCGGTTCTGGT	[GGC]_7_	285	FE523232
	R: AGGGAAGGCGTGAGTAGCG			
Primer150	F: AAGGAAGAGGAGGTGGAGAC	[GAT]_7_	170	FG616224
	R: TGCTTCTTCGCAAACCTG			
Primer166	F: AGAAGGGGTTAATGGAGAA	[GAG]_7_	126	DT638934
	R: TTCAGCAACCAACTTCTAAAT			
Primer184	F: ACTTGAATCAGTATCAAGGAGAGGA	[GGAGA]_5_	174	DT629297
	R: AGACTGGACGGCGACATAAAA			
Primer194	F: AGCATCAACAGGCACAGCAA	[CAG]_13_	260	DT625916
	R: AGCAGACCCACGCCCAAA			
Primer221	F: AGTTCGATTATCAAAATTCTGTATTGGC	[AAG]_6_	223	DT627258
	R: TTGGTTGGGGTGGTTCTGC			
Primer222	F: CGCCCTTAATTTCGCCCACT	[AAG]_6_	177	DT627469
	R: CATGAAGCCATCGTTCCCATAA			
Primer226	F: AAAGCCACCATTCACAGCA	[CAG]_6_	101	DT633646
	R: GTTTCTTGATAAAGATAAATCCCTC			
Primer243	F: CAAGGAGGAGATGTTGACAGGTT	[GAA]_6_	211	DT625793
	R: ATCTGAATCACGACCAACAACG			
Primer285	F: TCTGACCGATTTGTGCGA	[ACC]_9_	185	FE521917
	R: GGAAGAAGATACAGCGATATGA			

**Table 2:** Genetic parameters associated with each of the 14 EST-SSR primers.

Locus	Number of Alleles	*Ho*	*He*	*Fst*	*Nm*
Primer30	2	0.9419	0.4991	0.0010	251.4594
Primer33	6	0.5857	0.7176	0.0486	4.8943
Primer75	6	0.4093	0.7211	0.1324	1.6385
Primer89	5	0.0502	0.7301	0.0445	5.3729
Primer149	3	0.0149	0.2177	0.2861	0.6237
Primer150	5	0.0950	0.6472	0.1010	2.2260
Primer166	4	0.4038	0.5664	0.0832	2.7557
Primer184	5	0.2201	0.5071	0.2602	0.7108
Primer194	3	0.2313	0.2348	0.0529	4.4758
Primer221	3	0.2462	0.5750	0.0566	4.1652
Primer222	4	0.1680	0.6180	0.0701	3.3140
Primer226	3	0.4407	0.5016	0.0870	2.6234
Primer243	5	0.5226	0.7538	0.0230	10.6138
Primer285	2	0.3704	0.4938	0.2515	0.7442
Mean	4	0.3357	0.5570	0.0955	2.3672

Note: *Ho*, *He, F_ST_* and *Nm* are defined in Materials and Methods.

### Genetic parameters associated with samples from different species zones

Genetic parameters associated with samples from *P. massoniana*, *P. hwangshanensis* and contact zones are listed in Table 3. Among these parameters, *Na*, *Ne*, *I*, *Ho*, *He*, NPL and PPL indicate the degree of polymorphism, whereas *F_IS_* and *S* reflect the extent of hybridization among trees within each species zone. Values of the polymorphism-related parameters were highest for samples in the *P. massoniana* zone, and decreased as the transect approached the *P. hwangshanensis* zone. A similar variation trend was observed for *F_IS_* and *S* among the three zones, with values of these parameters clearly higher in the *P. massoniana* zone than in *P. hwangshanensis* and contact zones. The higher values indicate that hybridization is more frequent among trees within a given species zone than across zones. The high selfing rate consequently suggests that *P. massoniana* receives less pollen from outside zones than do hybrids and *P. hwangshanensis*. Comparisons of pairwise *F_ST_* values (Table 4) revealed that the lowest genetic differentiation was between *P. hwangshanensis* and contact zones (*F_ST_* = 0.0138). *F_ST_* was 0.1334 between *P. massoniana* and contact zones and 0.1516 between *P. massoniana* and *P. hwangshanensis* zones. An analysis of *Nm*, the parameter estimating gene flow across species zones, indicated that gene flow between *P. massoniana* and contact zones (*Nm* = 1.6241) was slightly higher than that between *P. massoniana* and *P. hwangshanensis* zones (*Nm* = 1.3991). Gene flow between the contact zone and the *P. hwangshanensis* zone (*Nm* = 17.8659), however, was significantly higher than for any other pairwise comparisons.

**Table 3:** Genetic parameters associated with samples collected from each species zone.

Collected No.	Elevation interval (m)	No. of samples	Na	Ne	I	H_o_	H_E_	NPL	PPL (%)	F_IS_	Selfing rate
*P. massoniana* zone	450–800	68	3.7857	2.6644	1.0325	0.3373	0.5717	53	94.64	0.417	0.5886
Contact zone	800–1050	57	3.6429	2.4114	0.9296	0.3329	0.5220	51	91.07	0.369	0.5391
*P. hwangshanensis* zone	1050–1820	145	3.5000	2.2977	0.8687	0.3363	0.4887	49	87.50	0.315	0.4791
Total	450–1820	270						56			

Note: *Na*, *Ne*, *I*, *Ho*, *He* and *F_IS_* are defined in Materials and Methods.

**Table 4:** Pairwise comparison of *F_ST_* and *Nm* across species zones.

F_ST_Nm	P. massoniana zone	Contact zone	P. hwangshanensis zone
The P. massoniana zone	-	0.1334	0.1516
The Contact zone	1.6241	-	0.0138
The P. hwangshanensis zone	1.3991	17.8659	-

### Bayesian admixture analysis and species boundary identification

**Figure 2:**
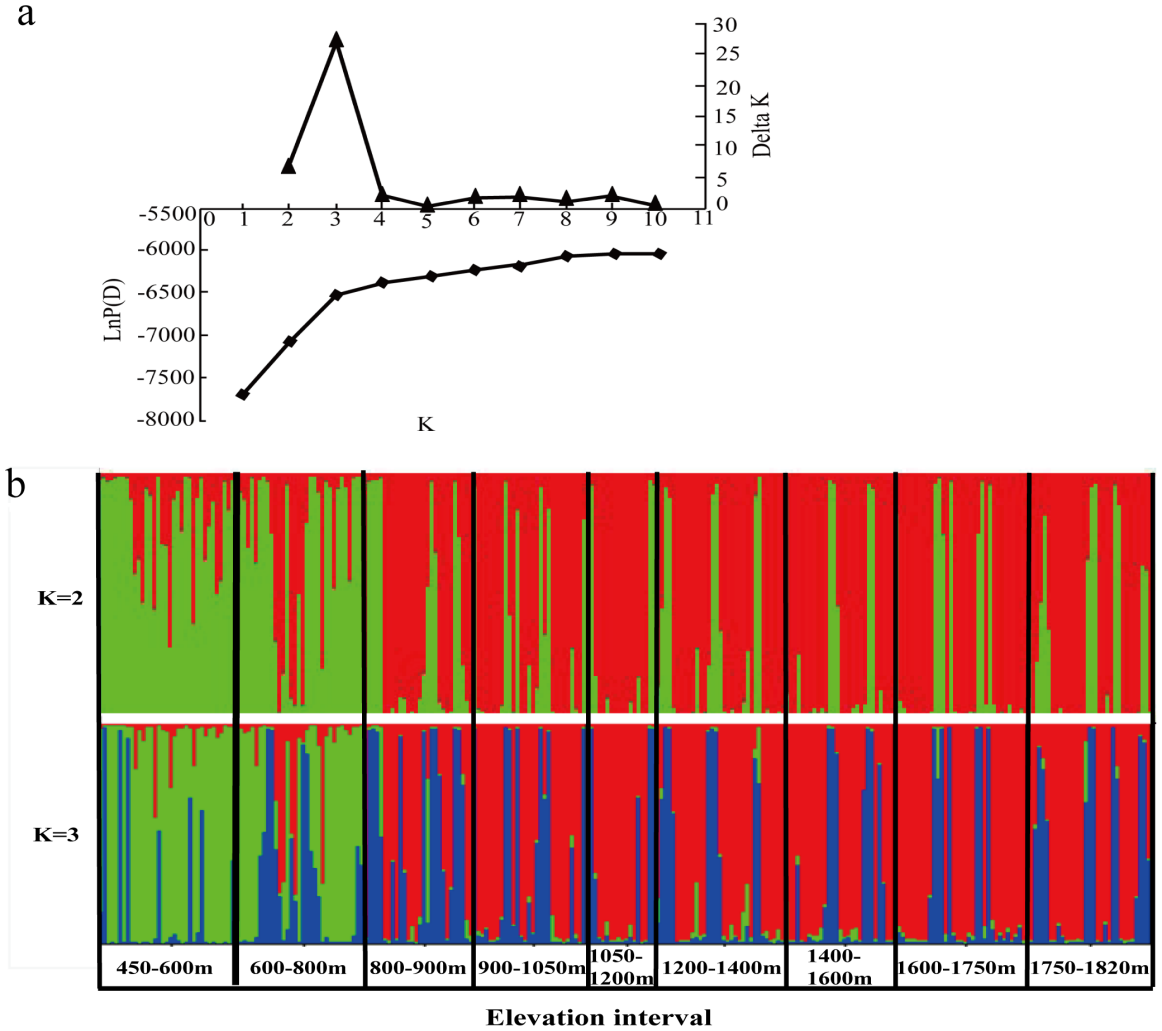
Bayesian admixture analysis of pines sampled within different elevation intervals. (**a**) Box-and-whisker diagram of ln *Pr*[*X*/*K*] for 10 runs at each *K* and *ΔK*, based on the rate of change of ln *Pr*[*X*/*K*] between successive *K* values. (**b**) Bayesian inference of population structure for *K* = 2 and *K* = 3. The elevation range was divided into nine intervals. Each interval contained approximately 30 samples and varied in size from 100 to 200 m depending on tree dispersal. Red, green and blue regions correspond to clusters 1, 2 and 3, respectively.

The likelihood of the partition of the data, ln *Pr*[*X*/*K*] increased sharply from *K* = 1 to *K* = 3, and then increased slightly from *K* = 3 to *K* = 10. Statistically, the optimal number of clusters (*K*) is determined based on the change in values of ln *Pr*[*X*/*K*] [32]. The partition of the data reached a plateau (Fig. 2a) at *K* = 3, which was indicated as the optimal number of clusters based on Δ*K* values. As demonstrated by Fig. 2b, cluster 1 was dominant at low elevation intervals (below 800 m) corresponding to the *P. massoniana* zone. Cluster 3 was mainly evident within elevation intervals associated with *P. hwangshanensis* and contact zones. Cluster 2 was widespread in both directions, and represented a population comprising backcrosses between hybrids and their parental species. This analysis indicated that direct gene introgression between *P. massoniana* and *P. hwangshanensis* is fairly low, but that backcrossing occurs intensively in both directions. Because there were only two parental populations in this study, we also performed the STRUCTURE analysis with *K* = 2. In both cases, the Bayesian admixture analysis clearly indicated that a distinguishable species boundary exists between *P. massoniana* and *P. hwangshanensis*. The uncovered boundary showed that the distribution of *P. massoniana* is constrained below 800 m, whereas the hybrids and *P. hwangshanensis* are distributed at higher elevations. The analysis at *K* = 2, however, only revealed the direct gene introgression between the two species. Previous studies have indicated that the distributions of *P. massoniana* and *P. hwangshanensis* overlap along a narrow hybrid zone, with trees in the hybrid zone possessing intermediate morphological and anatomical characteristics [14]–[16]. In our earlier studies, seed germination rates of trees in the hybrid zone were found to be significantly lower than those of trees in the parental species zones [22], [34]. Statistically, the optimal number of clusters was calculated as *K* = 3 rather than *K* = 2. We propose that the optimal *K* value of 3 implies the virtual existence of a hybrid zone. Natural hybrid zones have been observed in the distribution of many plant species [8], [9], [35] and play important roles in intermediating gene introgression between parental populations [7]. At *K* = 3, intensive backcrossing was inferred to occur in both directions, with an indistinct genetic boundary associated with the hybrid zone due to intensive introgression with both parental species. Compare with *P. massoniana*, *P. hwangshanensis* was found to backcross with the hybrids more intensively, in agreement with the empirical observation that morphological and anatomical characteristics of trees in the contact zone are biased towards *P. hwangshanensis* [23]. On the basis of a RAPD marker analysis, Luo and Zou [17] have proposed that gene flow is more intense from *P. massoniana* to *P. hwangshanensis*, contrary to the evidence of our study and previous reports [14], [16], [36]. Detailed examination revealed that gene flow into *P. hwangshanensis* is actually occurring mainly from the hybrids, not from *P. massoniana*. From a technical standpoint, SSR markers are more reliable and powerful than RAPD markers for population structure analysis [37].

### Variation in allele frequencies with elevation

**Figure 3:**
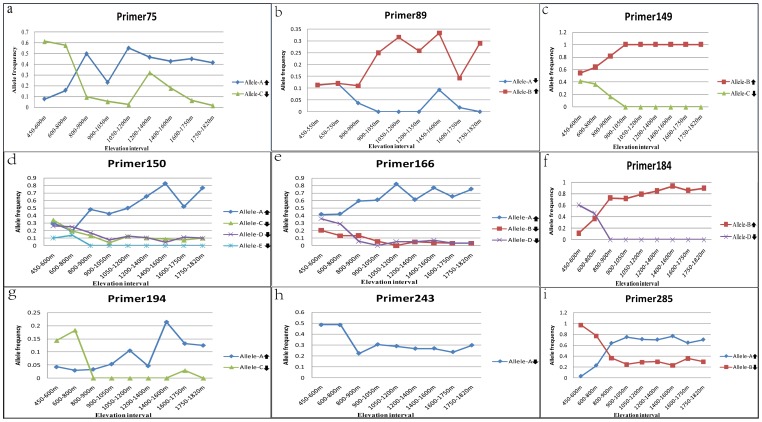
Variation in allele frequencies among different elevation intervals. (**a–i**) Genetic loci of different primer pairs. Alleles labeled with a down-arrow are type-II alleles, whose frequencies were negatively correlated with increasing elevation; alleles labeled with an up-arrow represent type-III alleles, whose frequencies were positively correlated with increasing elevation. The elevation range was divided as described in Fig. 2. Only type-II and type-III alleles generated by different primers are displayed; type-I alleles, whose frequencies were uncorrelated with elevation, are not shown.

We analyzed the frequencies of alleles generated by the different primer pairs along the divided elevation gradient. After excluding 11 rare alleles (frequency <0.1), the remaining 45 alleles were classified into three types according to their changes in frequency with increasing elevation. Frequencies of 25 alleles (55.56% of 45 alleles) were uncorrelated with elevation (type I; results not shown). We propose that the fitness of these alleles is hardly affected by elevation changes, allowing them to easily introgress into populations at different elevations. Frequencies of 12 alleles (26.67%; type II) were negatively correlated with increasing elevation. We hypothesize that these alleles are well-adapted to the *P. massoniana* zone; they are selected against at higher elevations, with their introgression hampered to differing extents when migrating towards the *P. hwangshanensis* zone. Among these type-II alleles, the frequencies of four (alleles C, E, D and C in Fig 3c, d, f and g, respectively) declined to zero in the elevation intervals above either 800 or 1,050 m, indicating that these alleles can not introgress into populations in the high-elevation species zones. Frequencies of the remaining eight alleles (17.78%) were positively correlated with increasing elevation (type III; Fig. 3). Type-III alleles correspond to alleles having good fitness in the *P. hwangshanensis* zone, but with their introgression blocked to some extent when dispersed towards the *P. massoniana* zone. As genetic introgression was mainly intermediated by backcrossing, our findings support the hypothesis proposed by Martinsen et al [7], that hybrid zones can act as genomic filters for selective gene introgression, thus maintaining the species boundary associated with type II and type III alleles. In this study, four alleles were found to be constrained only at low elevations, suggesting their potential application in the development of species-specific biomarkers.

## Discussion

Compared with genomic SSR markers EST-SSRs are more informative, as they represent portions of functional genes. Genetic introgression associated with EST-SSRs can be used to monitor the migration of transcribed genes across species. Nevertheless, SSR primers amplify multi-allelic loci, complicating efforts to record genotype data when analyzing samples from natural populations. We screened a large quantity of SSR primers in this study, selecting for use only those primers generating unambiguously scorable genotype profiles. When analyzed with these primers, parameters associated with polymorphism levels were generally found to decrease with increasing elevation. In this study, samples were collected within a defined distance to a transect line that was run from the foot to the top of the mountain. In the sampling area, *P. massoniana* mixes with broad-leaved trees and has a sparse on-site distribution. With increasing elevation, pure pine stands gradually dominate the forest landscape, with their on-site distribution fairly dense at high elevations. In our collections, the high-elevation samples thus tended to have closer kinship than the low-elevation ones, thereby leading to an underestimation of polymorphism at high elevations.

In this study, we monitored gene flow between *P. massoniana* and *P. hwangshanensis* along an elevation gradient on Huangshan Mountain. Because of its beautiful natural scenery, Huangshan Mountain was established as a natural conservation area in 1934. According to “The History of Huangshan Mountain” [38], pine in this area is a native forest component and the vegetation in this area is naturally regenerated. Pines are anemophilous plants. Gene flow in pine under natural conditions mainly occurs via pollen and seed dispersal. In general, pollen flow impacts genetic diversity within and among plant populations, whereas seed dispersal plays important roles in the colonization of new sites, the reestablishment of extinct populations, and local migration [39]. As a food resource, the cones of *P. massoniana* and *P. hwangshanensis* might be transferred by rodents across species zones. In pines, however, pollen flow overwhelms seed flow [40]–[42]. Thus, gene introgression between pine species is mainly attributed to pollen flow. The flowering phenology of *P. massoniana* and *P. hwangshanensis* overlaps from April to May [13]. Because *P. hwangshanensis* is distributed at high elevations, its pollen should theoretically be easily dispersed to the lower elevation species zone. A RAPD marker-based analysis by Luo and Zou, however, revealed that gene flow is more intense from *P. massoniana* to *P. hwangshanensis* [17]. Our study also demonstrated that *P. hwangshanensis* receives more outside pollen from the other species' zones than does *P. massoniana*, in agreement at least superficially with Luo and Zou's findings. Nevertheless, population structure analysis revealed that direct gene introgression between the two species is fairly low, with gene flow between *P. massoniana* and *P. hwangshanensis* mainly intermediated via backcrossing. As also indicated by an analysis of gene flow across species zones, genes introgressed into *P. hwangshanensis* were found to be mainly from the hybrid zone rather than directly from *P. massoniana*. Consequently, the conclusion that gene flow is more intense from *P. massoniana* to *P. hwangshanensis* is not supported by the results of our study.

*Pinus massoniana* and *P. hwangshanensis* are naturally distributed in different ecological niches. With increasing elevation, the environmental factors directly affecting plant growth and fitness, such as oxygen partial pressure, air temperature and moisture regime, soil temperature and water regime, and sunlight and ultraviolet light intensity, will also change. These ecological factors will exert selective pressure on internal factors to maintain species integrity. A study by Qu et al. [22] determined that seed plumpness, germination rates and weight per thousand seeds were significantly lower in trees in the contact zone than elsewhere, suggesting reduced hybrid fitness. Reduced hybrid fitness is sometimes the first indication of genetic incompatibilities that may ultimately lead to reproductive isolation and speciation [43]. In the study of some other pine species, findings also supported that some ecological factors (such as geography and environment) could help maintain and reinforce species differentiation and reproductive isolation [44], [45]. Such as *Pinus yunnanenis*, the geographical and environmental factors together created stronger and more discrete genetic differentiation, the discrete differentiation between two genetic groups is consistent with niche divergence and geographical isolation of these groups [44]. *Pinus densata* and its parental species have diverged in ecological preferences, some candidate ecological factors associated with habitat-specific adaptation were identified [45]. *Pinus massoniana* and *P. hwangshanensis* are distinguishable on the basis of morphological, cytological and timber anatomical characteristics. In this study, we identified a distinct species boundary between their natural distributional ranges. On the other hand, no distinct boundary was detected at the molecular level between hybrid and *P. hwangshanensis* zones (Fig. 2b). Empirical observation revealed that the contact zone spanned an approximate vertical range of 700–1,000 m that varied between different geographic locations [14], [15], [36]. Species zones revealed by molecular markers were inconsistent with the divisions deduced from empirical observations. Our study results provide strong evidence for intensive mutual gene introgression between the contact zone and the *P. hwangshanensis* zone, which has resulted in the merging of the two species zones. This finding is consistent with the observation that morphological and anatomical characteristics of trees in the contact zone are biased towards *P. hwangshanensis* [16], [23].

In this study, 44.44% of amplified alleles were either positively or negatively correlated with increasing elevation. Among these alleles, four were found to be blocked completely from the *P. hwangshanensis* zone, with introgression ability varying among the other alleles. In general, inner selective pressure is derived from genes that conserve the integrity of the species gene pool, contributing to reproductive barriers [46]. The different introgression abilities of the various alleles may be due to their functional alteration. However, differing numbers of repeats do not normally cause important phenotypic differences among expressed alleles. The impeded alleles are therefore less likely associated directly with the persistence of species integrity, and may simply be linked to selected genes. The genetic recombination distance of alleles and their linkage with genes under different selection pressures would affect allele efficiencies [47]. We developed SSR markers from transcribed genes, but none of the corresponding EST sequences were homologous to genes of known function. Thus, we cannot currently associate introgression abilities of amplified alleles with gene functions.

On the basis of morphological, anatomical, and molecular evidence, natural hybrids have been confirmed in the contact zone between *P. massoniana* and *P. hwangshanensis* [14], [17], [18]. Excessive introgressive hybridization can lead to genetic swamping and bring about extinction [4], [48], [49]. Barriers to hybridization and interspecific gene flow may thus be vital for the persistence of permanently adjacently distributed species that occur with interfertile hybrids. Hybrid zones can act as genomic sieves. The semi-permeability of species boundaries allows some alleles to pass freely between species while restricting those contributing to reproductive barriers [46]. The above hypothesis is supported by the example of Fremont cottonwood (*Populus fremontii*), narrowleaf cottonwood (*P. angustifolia*) and their hybrids [7]. Fremont cottonwood grows at elevations of approximately 1,300 to 1,500 m throughout the Weber River drainage in the U.S.A., while narrowleaf cottonwood grows at elevations of approximately 1,400 to 2,300 m. The two species overlap in a narrow contact zone marked by the occurrence of extensive hybridization. An RFLP analysis clearly indicated that that hybrid zone acts as a genomic filter for selective gene introgression. Our study has provided additional evidence to aid in the understanding of how adjacently distributed plants maintain species integrity in the face of naturally occurring hybridization.

## Conclusions

In this study, we resolved several major controversies arising from previous studies of speciation of two geographically overlapping pine species in the presence of natural hybrids. First, our results demonstrate that direct introgression between these two species is fairly low, with the majority of gene introgression intermediated through backcrossing. Although our findings were superficially in agreement with a previous conclusion that gene flow is more intense from *P. massoniana* to *P. hwangshanensis*, a more detailed examination revealed that gene introgression into *P. hwangshanensis* zone populations was mainly from the hybrid zone, with direct colonization from *P. massoniana* fairly rare. Second, our study uncovered a distinct boundary between the on-site distributions of *P. massoniana* and *P. hwangshanensis*. On the other hand, no distinct boundary was detected between the hybrid zone and the *P. hwangshanensis* zone. We found that intensive mutual gene introgression between the two species zones led them to merge with each other, consistent with the observation that morphological and anatomical characteristics of trees in the contact zone are biased towards *P. hwangshanensis*. Third, we determined that introgression ability varied among amplified alleles, with some completely blocked from the opposite species. Our study has provided additional evidence that hybrid zones can act as genomic sieves, thereby allowing neutral alleles to pass freely between species while restricting those contributing to reproductive barriers.

## Supporting Information

Supporting Information S1 The elevation and breast diameter of samples collected from Huangshan Mountain in this study.
(XLS)
